# Serum Trefoil Factor 3 in Endometrioid Endometrial Carcinoma: Exploratory Diagnostic Value and Association with Lymphovascular Space Invasion

**DOI:** 10.3390/jcm15176598

**Published:** 2026-08-26

**Authors:** Bilgesu Çetinel Kaygun, Varol Gülseren, Merve Özel Yetkin, Ertuğrul Şen, Gülden Başkol, Fulya Çağlı, Terlan Selin Kotan, İbrahim Serdar Serin, Bülent Özçelik, Kemal Güngördük

**Affiliations:** 1 Department of Obstetrics and Gynecology, School of Medicine, Izmir Katip Çelebi University, Izmir 35620, Turkey; 2 Division of Gynecologic Oncology, Department of Obstetrics and Gynecology, School of Medicine, Izmir Katip Çelebi University, Izmir 35620, Turkey; drvarolgulseren@gmail.com; 3 Department of Biochemistry, School of Medicine, Erciyes University, Kayseri 38030, Turkey; 4 Division of Gynecologic Oncology, Department of Obstetrics and Gynecology, School of Medicine, Erciyes University, Kayseri 38030, Turkey; 5 Department of Obstetrics and Gynecology, School of Medicine, Erciyes University, Kayseri 38030, Turkey; 6 Division of Gynecologic Oncology, Department of Obstetrics and Gynecology, Faculty of Medicine, Muğla Sıtkı Koçman University, Muğla 48000, Turkey

**Keywords:** endometrial cancer, leiomyoma, lymphovascular space invasion, trefoil factor family 3

## Abstract

**Objectives:** The primary objective of this exploratory study was to compare serum Trefoil factor family 3 (TFF3) levels among patients with grade 1–2 endometrioid endometrial cancer (EC), leiomyoma distorting the endometrial cavity, and healthy controls, and to explore the cross-sectional association between serum TFF3 and lymphovascular space invasion (LVSI) in EC. **Methods:** The prospective observational case–control study comprised three groups. The first group consisted of all grade I–II endometrioid-type EC patients who underwent hysterectomy at our institution. The second group comprised patients with leiomyoma causing compression in the endometrial cavity who also underwent hysterectomy. Patients with histopathological evidence of myoma after hysterectomy were included in the study. All patients who visited between January 2023 and January 2024 were enrolled in the first two groups. The third control group involved patients with normal findings on sonographic assessment who attended annual gynecological follow-up visits. **Results:** One hundred thirteen patients were enrolled in the study. Mean preoperative TFF3 levels were significantly higher in both EC and leiomyoma groups compared with controls, whereas EC and leiomyoma did not differ. Serum TFF3 decreased significantly on the first postoperative day in the EC group, whereas no significant change was observed in the leiomyoma group. Within the EC group, an exploratory comparison showed a significantly higher average TFF3 value in cases with lymphovascular space invasion (LVSI). **Conclusions:** Serum TFF3 is elevated in both benign and malignant pathologies involving the endometrial cavity and shows limited specificity for distinguishing EC from leiomyoma. The observed association with LVSI is preliminary and hypothesis-generating; larger, adequately powered studies are required before any diagnostic or prognostic role can be established.

## 1. Introduction

Endometrial adenocarcinoma (EC) is the most common cancer of the female reproductive system in the western world [1]. It is typically diagnosed in the early stages due to symptoms such as metrorrhagia or postmenopausal bleeding. The most common histological type is the endometrioid subtype [2]. Currently, there is no sufficiently accurate and convenient serum biomarker for the diagnosis and risk stratification of EC in routine clinical practice.

Recently, Trefoil factor family 3 (TFF3), a protein consisting of 59 amino acids that has been studied as a tumor marker in various cancers, can be measured in the serum [3]. TFF3 is a peptide associated with mucin in mucus-secreting glands and plays a protective role against environmental damage in the epithelial layer [4]. TFF3 is primarily expressed in goblet cells of the small intestine and colon, as well as in the uterus, breast, hypothalamus, pituitary gland, salivary gland, and respiratory tract [5]. Variations in TFF3 levels have been observed in several cancers, such as lung, colorectal, thyroid, and breast cancers, compared to normal levels [3,6,7,8,9]. We hypothesize that this protein, detected in the endometrium, may exhibit variations in endometrioid-type EC cases.

Leiomyomas are benign smooth-muscle neoplasms of the myometrium and are the most common uterine mesenchymal tumors [10,11]. Although they do not arise from the endometrium, cavity-distorting leiomyomas may alter endometrial architecture and local signalling, which could influence circulating biomarkers. Because systemic factors such as obesity, metabolic disease, inflammation and renal dysfunction may also affect serum biomarker concentrations, these factors should be considered when interpreting TFF3 levels [12].

To date, data comparing serum TFF3 levels among endometrioid EC, benign cavity-distorting leiomyoma, and healthy controls are limited, and evidence regarding its association with LVSI is scarce. The primary aim of this study was to compare serum TFF3 concentrations across these three groups. The secondary aim was to explore the cross-sectional association between serum TFF3 and lymphovascular space invasion (LVSI) in EC.

## 2. Materials and Methods

Patients who presented to the Gynecology and Obstetrics Clinic at Erciyes University between January 2023 and January 2024 were included in the study. The prospective observational case–control study comprised three groups. The first group consisted of patients with grade 1–2 endometrioid endometrial carcinoma who underwent hysterectomy at our institution. The second group comprised patients with leiomyoma causing compression in the endometrial cavity who also underwent hysterectomy. Patients with histopathological evidence of myoma after hysterectomy were included in the study. All patients who visited between January 2023 and January 2024 were enrolled in the first two groups. The third control group comprised consecutive women attending annual gynecological check-ups who had normal ultrasound findings and no active gynecological complaints. We used a dependent random sampling method to select the control group; these patients were matched to patients with endometrial cancer in terms of age and menopausal status. Exclusion criteria for the study encompassed the presence of other malignancies, use of alternative treatments such as radiotherapy and chemotherapy as primary interventions, and the existence of endocrine disorders (e.g., hypo/hyperthyroidism). Adjuvant radiotherapy or chemotherapy had not started when the last control TFF3 values of the patients who received adjuvant treatment were obtained. The study received ethical approval from the Erciyes University Clinical Research Ethics Committee (Decision no: 2022/787; date: 7 December 2022). All procedures were performed in accordance with the ethical standards of the institutional and/or national research committee and in compliance with the 1964 Helsinki Declaration and its subsequent revisions or similar ethical guidelines.

Clinical data, including patients’ ages, menopausal status, presence of additional comorbidities, medical histories, symptoms, heights, and weights were documented. All patients underwent an ultrasound examination during their initial visit. Written informed consent was obtained from all participants through the provision of consent form, which they signed voluntarily. Pathological analysis of patients in the EC group included an investigation of tumor type, size, depth of myometrial invasion, presence of lymphovascular space invasion (LVSI), cervical involvement, and lymph node (LN) status. Tumor staging was conducted according to the FIGO 2023 criteria [13]. Uterine specimens were collected from various parts, including the anterior and posterior aspects of the cervix, the lower uterine segment, and the uterine corpus. A minimum of six sections were sampled for each case, including the section exhibiting the most extensive tumor invasion observed during routine examination.

According to a study by Bignotti et al., a sample size of 37 patients was deemed sufficient to achieve a study power of 80% at a significance level of 0.05 [14].

Blood samples of approximately 6 mL were collected for TFF3 measurement (pg/mL) and centrifuged at 2000 rpm for 10 min. The resulting serum samples were used for analysis, and any surplus was aliquoted into Eppendorf tubes and stored at −80 °C until the day of analysis. Human TFF3 concentrations were measured using a commercially available sandwich ELISA kit (Catalog No. RE1622H; Reed Biotech Ltd., Shanghai, China) according to the manufacturer’s instructions. The assay has an analytical range of 78.13–5000 pg/mL with a lower limit of detection (sensitivity) of 46.88 pg/mL. According to the manufacturer’s specifications, the reported intra-assay coefficients of variation (CVs) were 6.98%, 5.21%, and 7.36%, whereas the inter-assay CVs were 3.10%, 3.62%, and 5.98%. Serum samples were analyzed without dilution and measured in single wells. Aliquots underwent one freeze–thaw cycle before ELISA measurement. Laboratory personnel performing the assays were blinded to the clinical group allocation of the samples.

All serum samples were obtained in the fasting state. In both hysterectomy groups, serum samples were obtained one day before surgery and 24 h postoperatively. Blood samples from the control group were obtained during routine gynecological check-ups at the outpatient clinic; no postoperative sample was applicable in this group, as these women did not undergo surgery. The comparison of TFF3 values in the preoperative and postoperative periods for patients undergoing hysterectomy was conducted using the paired-*t* test.

Categorical variables are summarized as frequencies and percentages and were compared using the chi-square test. Distributional assumptions were evaluated using the Shapiro–Wilk test. For normally distributed variables, one-way ANOVA was used; for non-normally distributed variables, the Kruskal–Wallis test was applied. Where the omnibus test indicated a significant difference among the three groups, pairwise post-hoc comparisons were performed. Homogeneity of variance was assessed using Levene’s test, and the post-hoc procedure was selected accordingly. Normally distributed variables are presented as mean ± SD, whereas non-normally distributed variables are presented as median (min–max). Comparisons within dependent groups were performed using the paired *t*-test. ROC analysis was used to estimate cut-off values, sensitivity, specificity and area under the curve (AUC). The cut-off value was calculated using the bootstrapping method. The primary analysis was the comparison of serum TFF3 concentrations across the three study groups. Analyses of clinicopathological variables within the EC group were secondary and exploratory, and Bonferroni correction was applied to this family of comparisons. For four exploratory clinicopathological comparisons, the Bonferroni-adjusted significance threshold was *p* < 0.0125. Logistic regression was used to define factors predictive of endometrial cancer, and the results are presented as odds ratios (OR) with 95% confidence intervals (CI). No regression model was fitted for lymphovascular space invasion, as only seven patients had substantial LVSI and the number of events was insufficient to support regression estimation. Data recording and statistical analyses were performed using SPSS (Statistical Package for The Social Sciences) software (version 17, SPSS, Inc., Chicago, IL, USA). A *p*-value of <0.05 was considered to indicate statistical significance. The study was reported in accordance with the STROBE statement (Supplementary Material S1).

## 3. Results

A total of 113 patients were enrolled in the study. Thirty-two patients had EC, 37 patients underwent hysterectomy due to cavity-distorting leiomyoma, and 44 patients with sonographically normal genital structures were included as controls. The clinical and demographic data of the patients are presented in Table 1. No significant differences were observed among the groups in terms of patients’ age, menopausal status, and hemoglobin levels. Additionally, there were no differences in the prevalence of hypertension and diabetes when chronic diseases were assessed among the groups.

When body mass index was compared among the three groups using a post hoc Bonferroni test, the pairwise comparisons showed a significant difference between endometrial cancer and leiomyoma (*p* = 0.005), a significant difference between endometrial cancer and the control group (*p* < 0.001), but no significant difference between the leiomyoma group and the control group (*p* = 0.547). Because obesity is an established risk factor for endometrioid endometrial carcinoma, body mass index was entered together with TFF3 into the multivariable logistic regression model presented in Table 2, in which TFF3 remained independently associated with endometrial cancer after adjustment for body mass index and the other covariates.

The parameters used to predict endometrial cancer and the regression analysis are given in Table 2. The aim of this analysis was to identify variables distinguishing endometrial cancer patients from leiomyoma and control patients.

When evaluating the TFF3 values obtained in the initial assessment, a significant difference was noted among the groups, with the EC group showing values of 3898 ± 1436 pg/mL (95% CI 3380–4416), the leiomyoma group showing values of 3574 ± 2216 pg/mL (95% CI 2835–4313), and the control group showing values of 1350 ± 1765 pg/mL (95% CI 813–1887) (*p* < 0.001). In the post-hoc Bonferroni test, significant differences were found between the EC group and the control group (*p* < 0.001) as well as between the leiomyoma group and the control group (*p* < 0.001). No significant difference was observed between the EC and leiomyoma groups (*p* = 1.0). In the EC group, serum TFF3 decreased significantly on the first postoperative day, from 3898 ± 1436 pg/mL (95% CI 3380–4416) to 3425 ± 1019 pg/mL (95% CI 3057–3792) (*p* = 0.030). In the leiomyoma group, no significant change was observed, with values of 3574 ± 2216 pg/mL (95% CI 2835–4313) preoperatively and 3531 ± 2252 pg/mL (95% CI 2780–4282) on the first postoperative day (*p* = 0.801).

**Figure 1:**
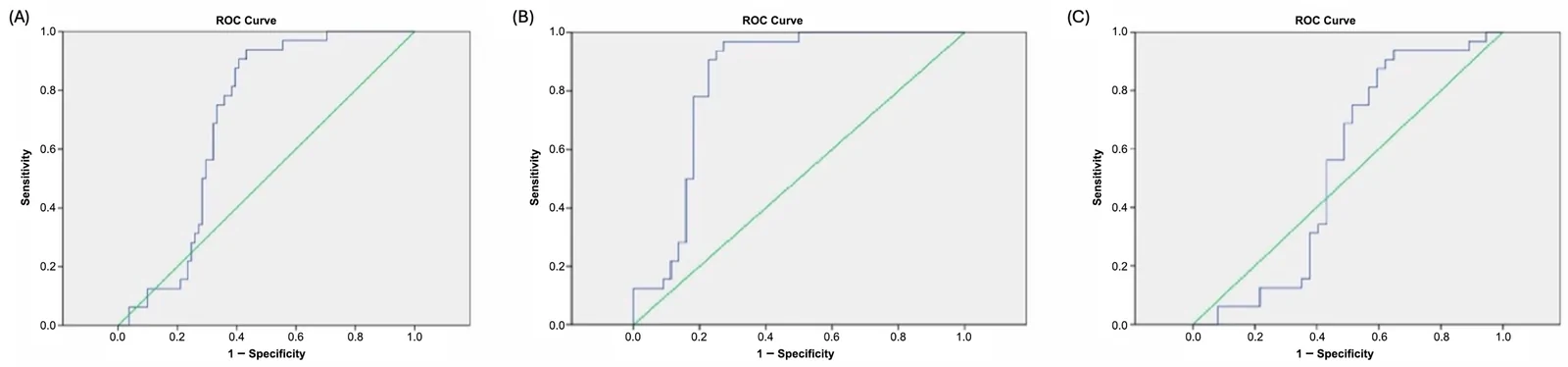
(**A**): Receiver operating characteristic (ROC) curve for serum TFF3 in distinguishing endometrioid endometrial carcinoma (EC) from non-EC participants (leiomyoma and healthy controls) (*p* = 0.001). The area under the curve (AUC) was 0.701 (95% CI 0.607–0.795). A cut-off value of 2406 pg/mL yielded a sensitivity of 90.6% (95% CI 75.0–98.0) and a specificity of 59.3% (95% CI 47.8–70.1). (**B**): ROC for TFF3 in distinguishing EC from healthy controls (*p* < 0.001). The AUC was 0.835 (95% CI 0.739–0.931). A cut-off value of 1406 pg/mL yielded a sensitivity of 96.9% (95% CI 83.8–99.9) and a specificity of 72.7% (95% CI 57.2–85.0). (**C**): ROC for TFF3 in distinguishing EC from leiomyoma (*p*: not applicable). The AUC was 0.541 (95% CI: NA). A cut-off value is not applicable.

To explore the diagnostic discrimination of TFF3 for EC versus non-EC participants (leiomyoma patients and healthy controls combined) at the time of diagnosis, a ROC analysis was performed (Figure 1A). The calculated AUC was 0.701 (95% CI = 0.607–0.795). At a TFF3 cut-off value of 2406 pg/mL, the sensitivity was 90.6% (95% CI 75.0–98.0) and the specificity was 59.3% (95% CI 47.8–70.1) (Youden’s Index: 49.6), indicating only modest discrimination and confirming that serum TFF3 alone is unlikely to be clinically useful as a standalone diagnostic biomarker. A second ROC analysis was performed between EC and healthy control patients (Figure 1B), which yielded an AUC of 0.835 (95% CI 0.739–0.931; *p* < 0.001). A cut-off value of 1406 pg/mL yielded a sensitivity of 96.9% (95% CI 83.8–99.9) and a specificity of 72.7% (95% CI 57.2–85.0) (Youden’s Index: 69.6). In Figure 1C, an attempt was made to determine a TFF3 cut-off value to differentiate EC patients from leiomyoma patients. However, the area under the curve was insufficient, the comparison did not reach statistical significance, and no cut-off value was applicable. Sensitivity and specificity could not be determined for this comparison.

Within the EC group, average TFF3 values were compared based on the presence of histopathological prognostic factors (Table 3). It was observed that higher TFF3 values were present in cases with deep myometrial invasion (4662 ± 2004 pg/mL) compared to those with superficial invasion (3643 ± 1135 pg/mL), although this difference did not reach statistical significance (*p* = 0.082). Furthermore, a significantly higher average TFF3 value was noted in cases with substantial LVSI (5304 ± 1382 pg/mL vs. 3504 ± 1204 pg/mL; *p* = 0.002). This association remained significant after Bonferroni correction, but it is based on only seven patients with substantial LVSI and is therefore exploratory and should be regarded as preliminary.

No regression model was fitted for the presence of substantial LVSI. Only seven patients had substantial LVSI, and the number of events was insufficient to support regression estimation; no inference regarding independent predictors of LVSI is therefore drawn.

## 4. Discussion

The study investigated the potential of the TFF3 protein, previously reported to be expressed in the endometrium, as a marker in EC cases. It was determined that the TFF3 level was higher in the malignant EC and benign leiomyoma groups compared to healthy individuals. However, no significant difference in TFF3 levels was observed between EC patients and benign leiomyoma patients. Notably, TFF3 levels were significantly higher in EC patients with LVSI.

Although TFF3 has been reported to be expressed in the uterus, its precise function remains unclear [15]. TFFs play a crucial role in maintaining the surface integrity of the mucosal epithelium and are involved in proliferation, apoptosis, cell migration, and angiogenesis [5,7,9]. Notably, TFF3 does not fluctuate during the menstrual cycle phases [4,16]. Evidence shows that TFF3 is implicated in embryo implantation and endometrial receptivity [9,17]. A study by Neubert et al. reported elevated TFF3 levels in postmenopausal women with endometrial cancer compared to those with a normal endometrium [18]. This trend of higher TFF3 concentrations was also observed in endometrial cancer cases relative to patients with hyperplasia and normal endometrium [14,19]. The study by Bignotti et al., which included 25 endometrial cancer cases, 13 endometrial hyperplasia cases, and 47 control patients, demonstrated that with a cut-off of 752 ng/mL, TFF3 had a sensitivity of 56% and specificity of 85% in distinguishing EC from hyperplasia, as well as a specificity of 71% in distinguishing hyperplasia from normal endometrium [14]. The absolute values reported in that study are two to three orders of magnitude higher than those measured here. The information available to us does not allow us to determine the reason for this difference. The two studies used different immunoassays, and the cut-off value reported by Bignotti et al. lies well outside the analytical range of the assay used in the present study, as stated in the Methods; the two sets of values are therefore not directly comparable in absolute terms. Absolute serum TFF3 concentrations, and any cut-off derived from them, should be regarded as specific to the assay employed. The cut-off values reported here apply only to the kit described in the Methods, and comparison between studies should be limited to the direction of between-group differences rather than to absolute concentrations. Immunostaining results revealed a TFF3 positivity rate of 75% for endometrioid-type cancer, 12% for serous cancer, and 19% for clear cell carcinomas (*p* < 0.001) [20]. Additionally, TFF3 levels were significantly higher with increasing grades of EC (3.3 ± 0.2 pg/mL for grade 1, 3.6 ± 0.3 pg/mL for grade 2, 4.3 ± 0.3 pg/mL for grade 3; *p* = 0.01) [21]. Consistent with Neubert et al. and Bignotti et al., we observed higher TFF3 levels in EC compared with controls; however, our data additionally show that leiomyomas distorting the cavity can similarly elevate serum TFF3. This suggests that serum TFF3 may be elevated in both malignant and benign pathology involving the endometrial cavity. Myomas outside the endometrium exert pressure on the endometrium, leading to changes in vascularity and receptivity and triggering the production of various proteins (including matrix metalloproteinases 2–11, endothelial growth factor, basic fibroblast growth factor, heparin-binding epidermal growth factor, platelet-derived growth factor, and parathyroid hormone-related protein) [12]. Consequently, our findings imply that serum TFF3 concentrations may be indirectly affected by such conditions.

Serum TFF3 was elevated in both groups with pathology involving the endometrial cavity, namely endometrioid carcinoma arising from the endometrium itself and leiomyoma distorting the cavity. In contrast, serum TFF3 was significantly lower in women with a sonographically normal endometrial cavity and no gynecological complaint. As all three groups had an intact uterus at the time of sampling, this pattern cannot be attributed to the presence of the organ itself and instead indicates that pathology involving the endometrial cavity, whether malignant or benign, is associated with elevated serum TFF3. We emphasize that this is an association and not a statement about the tissue of origin of circulating TFF3; our study measured serum concentrations only and provides no data on the site of production. Serum TFF3 decreased significantly on the first postoperative day in the EC group, whereas no significant change was observed in the leiomyoma group. Since both groups underwent the same operation, this pattern does not readily support a purely non-specific perioperative explanation, such as hemodilution or the acute phase response, for the change observed in the EC group. We would emphasize, however, that we did not formally compare the magnitude of the change between the two groups, and a difference in statistical significance between them should not in itself be taken as evidence that the changes differ. Two further considerations limit what can be inferred from these measurements. First, mean postoperative values in both groups remained well above the mean value of the control group, so no normalization occurred within the first 24 h; second, since a clear half-life for TFF3 has not been determined in the literature, and the limited budget of our study did not permit sampling beyond postoperative day 1, the subsequent course of these values is unknown. We therefore regard the postoperative measurements as consistent with, but not confirmatory of, the association described above. The potential longitudinal behavior of serum TFF3 requires evaluation in specifically designed follow-up studies.

When the TFF3 cut-off value was set at 2406 pg/mL, it demonstrated a sensitivity of 90.6% and a specificity of 59.3% for EC. Furthermore, the presence of LVSI was associated with significantly higher average TFF3 values (5304 ± 1382 pg/mL vs. 3504 ± 1204 pg/mL; *p* = 0.002). Although higher TFF3 values were observed in cases with deep myometrial invasion, this result was not statistically significant. Because only seven patients had substantial LVSI, this association should be regarded as exploratory and hypothesis-generating. The number of events was insufficient to support regression modeling, and we therefore make no claim as to whether serum TFF3 is independently associated with LVSI. Given its limited specificity for EC over leiomyoma, serum TFF3 alone is unlikely to be clinically useful as a standalone diagnostic biomarker.

Previous studies have shown that TFF3 activates estrogen receptor alpha, thereby contributing to antiestrogen resistance [8]. Additionally, tamoxifen treatment has been demonstrated to increase the expression of the ER-sensitive TFF3 gene, supporting the development of EC [20]. TFF3 is likely to contribute to cancer formation by activating ERK1/2, a critical component of the MAPK signaling pathway, and stimulating tumor cell proliferation [5]. Consequently, the potential use of a TFF3 inhibitor in the treatment of EC warrants investigation through prospective studies.

TFF3 has been increasingly investigated as a circulating biomarker in gynecologic malignancies; however, its clinical utility remains less established compared to more widely studied markers such as HE4 and CA125. HE4 has demonstrated superior diagnostic performance and correlation with high-risk clinicopathological features in endometrial cancer, whereas CA125 retains clinical relevance in advanced-stage disease and extrauterine spread [22]. In addition, systemic inflammatory markers and emerging approaches such as circulating tumor DNA have shown promise in reflecting tumor burden and biological aggressiveness [23]. In this context, our findings suggest that although TFF3 is elevated in both benign and malignant endometrial conditions, its limited specificity restricts its role as a standalone diagnostic biomarker. Instead, TFF3 may have potential value when interpreted alongside established markers within multimodal predictive models [24,25].

The observed association between elevated serum TFF3 levels and LVSI may be biologically plausible. TFF3 is known to play a role in epithelial repair, cell migration, and anti-apoptotic signaling, processes that may also facilitate tumor cell dissemination [26]. Experimental evidence suggests that TFF3 may contribute to tumor progression through modulation of epithelial–mesenchymal transition, enhancement of cellular motility, and interaction with mucin-related pathways [27]. These mechanisms could promote intravasation and vascular spread, providing a potential link to LVSI. Recent studies focusing on prognostic stratification in endometrial cancer, including models incorporating clinicopathological factors, molecular classification, and imaging parameters, emphasize the importance of identifying markers associated with aggressive disease behavior and LVSI [28,29,30]. In this regard, our findings should be interpreted as hypothesis-generating and warrant further validation in larger cohorts integrating molecular and serum biomarkers.

### Limitations and Strengths

This was a single-center study with a relatively small sample size and limited follow-up. Several limitations should be emphasized. First, only grade 1–2 endometrioid EC cases were included; therefore, the findings cannot be generalized to high-grade endometrioid, serous, clear-cell or other histological subtypes. Second, although 37 patients per group were planned, only 32 EC patients were enrolled, which may have reduced statistical power, particularly for subgroup analyses and non-significant comparisons. Third, body mass index differed significantly among the groups. Although TFF3 remained independently associated with endometrial cancer after multivariable adjustment for body mass index, the groups were not matched on body mass index at enrollment and residual confounding by adiposity cannot be excluded. Fourth, some pathological subgroups were extremely small, including only seven LVSI-positive cases, five node-positive cases and one case with cervical involvement, so these findings must be interpreted cautiously. The small number of LVSI events also precluded regression modeling, so the observed association between serum TFF3 and substantial LVSI is unadjusted for tumor grade, depth of myometrial invasion or other clinicopathological factors and could be confounded by them. Fifth, postoperative TFF3 was measured only once, at 24 h, and the magnitude of the change was not formally compared between the two surgical groups. The postoperative findings should therefore be regarded as supportive rather than confirmatory of the association between endometrial cavity pathology and elevated serum TFF3. Finally, absolute TFF3 values are assay-dependent and cannot be compared numerically with those of studies using different immunoassays, which is an additional methodological limitation. Nonetheless, the originality of comparing EC with both leiomyoma and healthy controls remains a strength.

## 5. Conclusions

In conclusion, serum TFF3 is elevated in both benign and malignant pathologies involving the endometrial cavity and has limited specificity for distinguishing EC from leiomyoma. Both study groups were characterized by involvement of the endometrial cavity, directly in endometrioid carcinoma and indirectly through cavity distortion in the leiomyoma group. Our findings therefore describe an association between such pathology and circulating TFF3 concentrations rather than the tissue of origin of the peptide. The observed association with LVSI should be considered preliminary and exploratory. Larger, adequately powered, preferably multicenter studies with standardized assay reporting, clinically relevant comparator groups, multivariable adjustment and longitudinal outcomes are needed before any diagnostic, prognostic or follow-up role can be established.

## Figures and Tables

**Table 1:** Demographic characteristics of the study group.

	Endometrial Cancer (*n* = 32)	Leiomyoma (*n* = 37)	Control (*n* = 44)	*p*
Age (years) ^a^	62.5 ± 8.4 (59.2–65.2)	58.3 ± 10.5 (54.7–61.8)	62.3 ± 8.0 (59.9–64.8)	0.091
Postmenopausal status, *n* (%)	28 (87.5%)	27 (73.0%)	40 (90.9%)	0.074
Body mass index (kg/m^2^) ^a^	29.4 ± 4.4 (27.8–31.0)	27.1 ± 2.7 (26.2–28.0)	26.2 ± 1.4 (25.8–26.7)	<0.001
Hemoglobin (g/dL) ^a^	11.4 ± 0.8 (11.1–11.7)	11.6 ± 0.7 (11.3–11.8)	11.5 ± 0.8 (11.2–11.7)	0.736
Hypertension, *n* (%)	13 (40.6%)	10 (27.0%)	7 (15.9%)	0.055
Diabetes Mellitus, *n* (%)	11 (34.4%)	10 (27.0%)	7 (15.9%)	0.170

^a^ mean ± standard deviation (95% confidence interval). Post hoc pairwise comparisons for body mass index (Bonferroni): EC vs. leiomyoma, *p* = 0.005; EC vs. control, *p* < 0.001; leiomyoma vs. control, *p* = 0.547.

**Table 2:** Regression analysis with parameters used to predict endometrial cancer.

	Univariable			Multivariable		
	OR	95% CI	*p*	OR	95% CI	*p*
Trefoil factor family 3	1.1	1.1–1.1	0.001	1.1	1.1–1.1	0.013
Body mass index	1.3	1.1–1.5	0.001	1.2	1.1–1.5	0.003
Age	1.0	0.9–1.1	0.362	0.9	0.8–1.1	0.769
Postmenopausal status	1.4	0.4–4.8	0.533	3.2	0.2–36.6	0.349
Hemoglobin (g/dL)	0.8	0.5–1.4	0.592	1.0	0.5–1.9	0.830
Hypertension	2.5	1.1–6.2	0.036	3.5	0.8–10.7	0.063
Diabetes Mellitus	1.9	0.7–4.8	0.141	1.2	0.4–3.8	0.642

OR: Odds Ratio, CI: Confidence Interval.

**Table 3:** Comparison of prognostic factors with TFF3 values in endometrial cancer patients.

	TFF3 (pg/mL)	*p*
Grade ^a^		0.737
1 (*n* = 12)	4011 ± 1689 (2937–5085)	
2 (*n* = 20)	3830 ± 1304 (3220–4441)	
Myometrial invasion ^a^		0.082
Superficial (<1/2) (*n* = 24)	3643 ± 1135 (3164–4123)	
Deep (≥1/2) (*n* = 8)	4662 ± 2004 (2986–6337)	
Cervical involvement ^a^		
Positive for involvement (*n* = 1)	4635	
Negative for involvement (*n* = 31)	3874 ± 1454 (3341–4408)	
Lymphovascular space invasion ^a^		0.002
Negative (*n* = 25)	3504 ± 1204 (3007–4001)	
Substantial (*n* = 7)	5304 ± 1382 (4026–6582)	
Lymph node ^a^		0.659
Negative for metastasis (*n* = 27)	3947 ± 1259 (3449–4445)	
Positive for metastasis (*n* = 5)	3632 ± 2363 (697–6567)	

^a^ mean ± standard deviation (95% confidence interval) Cervical involvement was not tested, as only one patient was positive.

## Data Availability

The datasets generated and/or analyzed during the current study are not publicly available due to patient data safety restrictions but are available from the corresponding author on reasonable request.

## Supplementary Materials

The following supporting information can be downloaded at: https://www.mdpi.com/article/10.3390/jcm15176598/s1, Supplementary Material S1: STROBE Statement—Checklist of items that should be included in reports of case–control studies.
