# Supplementary material for: Serum Trefoil Factor 3 in Endometrioid Endometrial Carcinoma: Exploratory Diagnostic Value and Association with Lymphovascular Space Invasion
Source: J Clin Med. 2026 Aug 26;15(17):6598. doi: 10.3390/jcm15176598 (PMC13566039; doi:10.3390/jcm15176598)
Supplement: Supplementary file 1 [file jcm-15-06598-s001.zip › Supplementary Material S1.pdf]

STROBE Statement—Checklist of items that should be included in reports of case–control studies

Serum trefoil factor 3 in endometrioid endometrial carcinoma: exploratory diagnostic value and association with lymphovascular space invasion

Item	*Recommendation*	*Reported*	*Location in Manuscript*
1a	Indicate the study design with a commonly used term in the title or the abstract	Yes	Title; Abstract – Methods ("prospective observational case/control study")
1b	Provide in the abstract an informative and balanced summary of what was done and what was found	Yes	Abstract (structured: Objective, Methods, Results, Conclusion)
2	Explain the scientific background and rationale for the investigation	Yes	Introduction
3	State specific objectives, including any prespecified hypotheses	Yes	End of Introduction; Abstract – Objective
4	Present key elements of study design early in the paper	Yes	Material and Method (first paragraph)
5	Describe the setting, locations, and relevant dates, including periods of recruitment, exposure, follow-up, and data collection	Yes	Material and Method
6a	Give the eligibility criteria, and the sources and methods of case ascertainment and control selection. Give the rationale for the choice of cases and controls	Yes	Material and Method (three-group definition; inclusion / exclusion criteria)
6b	For matched studies, give matching criteria and the number of controls per case	Yes	Material and Method
7	Clearly define all outcomes, exposures, predictors, potential confounders, and effect modifiers. Give diagnostic criteria, if applicable	Yes	Material and Method (serum TFF3 as exposure; EC/LVSI outcomes; clinical & pathological variables)
8	For each variable of interest, give sources of data and details of methods of assessment (measurement). Describe comparability of assessment methods if there is more than one group	Yes	Material and Method (serum TFF3 by sandwich ELISA; kit catalogue, assay range, intra-/inter-assay CVs)
9	Describe any efforts to address potential sources of bias	Partial	Material and Method (assay not blinded to group; single freeze–thaw noted); Limitations (selection bias, confounding by BMI)
10	Explain how the study size was arrived at	Yes	Material and Method (sample size from Bignotti et al.; 80% power at $\alpha = 0.05$; 37 per group)
11	Explain how quantitative variables were handled in the analyses. If applicable, describe which groupings were chosen and why	Yes	Statistical Analysis (mean ± SD / median (min–max); ROC-derived cut-offs)
12a	Describe all statistical methods, including those used to control for confounding	Yes	Statistical Analysis (chi-square, ANOVA, Kruskal–Wallis, paired-t, logistic regression, ROC)
12b	Describe any methods used to examine subgroups and interactions	Yes	Results (prognostic-factor subgroups within the EC group, Table 3)
12c	Explain how missing data were addressed	No	Not described
12d	If applicable, explain how matching of cases and controls was addressed	N/A	No analytic adjustment for matching reported
12e	Describe any sensitivity analyses	No	Not performed

Item	*Recommendation*	*Reported*	*Location in Manuscript*
13a	Report the numbers of individuals at each stage of the study – e.g., numbers potentially eligible, examined for eligibility, confirmed eligible, included in the study, completing follow-up, and analysed	Yes	Results (113 enrolled: 32 EC, 37 leiomyoma, 44 controls)
13b	Give reasons for non-participation at each stage	No	Not reported
13c	Consider use of a flow diagram	No	Not included
14a	Give characteristics of study participants (e.g., demographic, clinical, social) and information on exposures and potential confounders	Yes	Table 1; Results (demographic and clinical characteristics)
14b	Indicate the number of participants with missing data for each variable of interest	No	Not tabulated
15	Report numbers in each exposure category, or summary measures of exposure	Yes	Results; Tables 1–4 (serum TFF3 values per group and by prognostic factor)
16a	Give unadjusted estimates and, if applicable, confounder-adjusted estimates and their precision (e.g., 95% CI). Make clear which confounders were adjusted for and why they were included	Yes	Results (logistic regression odds ratios with 95% CI, Tables 2 and 4)
16b	Report category boundaries when continuous variables were categorized	Yes	Results
16c	If relevant, consider translating estimates of relative risk into absolute risk for a meaningful time period	N/A	Not applicable
17	Report other analyses done – e.g., analyses of subgroups and interactions, and sensitivity analyses	Yes	Results (ROC analyses Figures 1A–1C; pre-/postoperative paired comparison; prognostic subgroups)
18	Summarise key results with reference to study objectives	Yes	Discussion (opening paragraph)
19	Discuss limitations of the study, taking into account sources of potential bias or imprecision. Discuss both direction and magnitude of any potential bias	Yes	Discussion – Limitations and strengths
20	Give a cautious overall interpretation of results considering objectives, limitations, multiplicity of analyses, results from similar studies, and other relevant evidence	Yes	Discussion; Conclusion
21	Discuss the generalisability (external validity) of the study results	Yes	Discussion – Limitations (single-centre; grade 1–2 endometrioid only)
22	Give the source of funding and the role of the funders for the present study and, if applicable, for the original study on which the present article is based	Yes	Funding statement; Conflict of interest; Acknowledgments

Note: An Explanation and Elaboration article discusses each checklist item and gives methodological background and published examples of transparent reporting. The STROBE checklist is best used in conjunction with this article (freely available on the Web sites of PLoS Medicine at http://www.plosmedicine.org/, Annals of Internal Medicine at http://www.annals.org/, and Epidemiology at http://www.epidem.com/). Information on the STROBE Initiative is available at http://www.strobe-statement.org.
